# *catena*-Poly[[diaqua­strontium]-bis­(μ-2-bromo­benzoato)-κ^2^ *O*,*O*′:*O*′;κ^3^ *O*:*O*,*O*′]

**DOI:** 10.1107/S1600536809045395

**Published:** 2009-11-04

**Authors:** Bi-Song Zhang

**Affiliations:** a College of Material Science and Chemical Engineering, Jinhua College of Profession and Technology, Jinhua, Zhejiang 321017, People’s Republic of China

## Abstract

The hydro­thermal reaction of SrCO_3_ and 2-bromo­benzoic acid in CH_3_OH–H_2_O afforded the Sr^II^ title polymeric complex, [Sr(C_7_H_4_BrO_2_)_2_(H_2_O)_2_]_*n*_. Within the coordination sphere, the Sr^II^ ion is located on a crystallographic twofold axis, and is coordinated by eight O atoms from two water mol­ecules and four carboxyl­ate groups of 2-bromo­benzoate ligands in an irregular coordination geometry. Two μ_3_-carboxyl­ate groups of the 2-bromo­benzoate anions bridge two symmetry-related Sr^II^ atoms, giving rise to a chain structure extending along [001]. The polymeric chains are connected *via* O—H⋯O and O—H⋯Br hydrogen bonds inter­actions into a three-dimensional supra­molecular network.

## Related literature

For other metal complexes with the 2-bromo­benzoato ligand, see: Zhang *et al.* (2005 ▶, 2008 ▶); Zhang (2006 ▶); Wang *et al.* (2003 ▶). For related structures, see: Zhang (2008 ▶); Karipides *et al.* (1988 ▶).
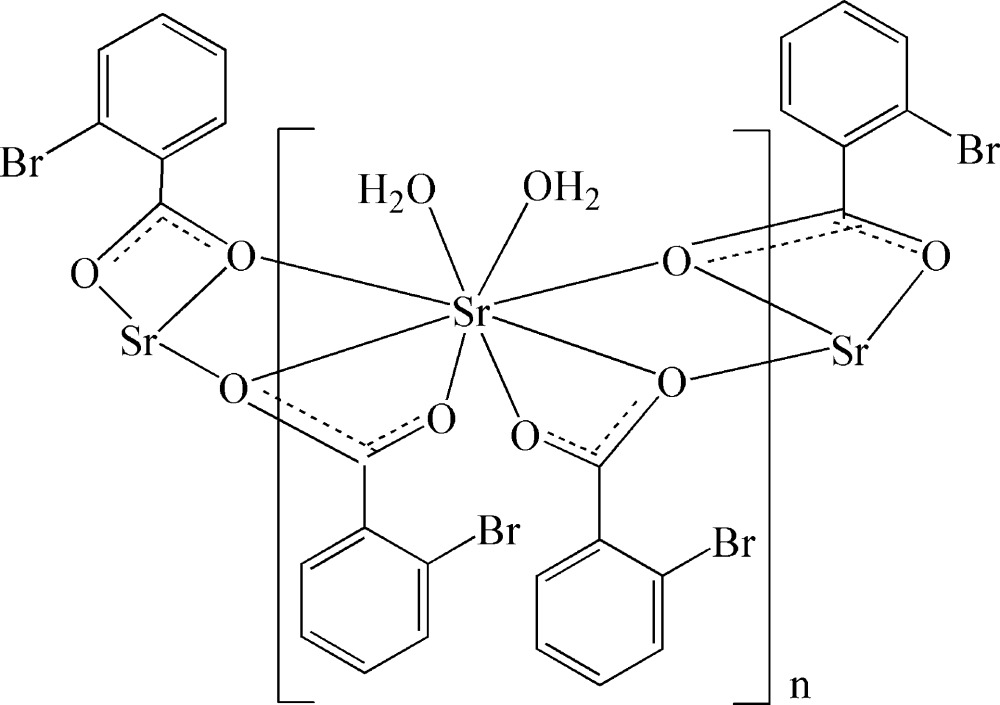


## Experimental

### 

#### Crystal data

[Sr(C_7_H_4_BrO_2_)_2_(H_2_O)_2_]
*M* *_r_* = 523.68
Orthorhombic, 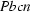

*a* = 18.740 (4) Å
*b* = 11.669 (2) Å
*c* = 8.0529 (16) Å
*V* = 1760.9 (6) Å^3^
*Z* = 4
Mo *K*α radiation
μ = 7.62 mm^−1^
*T* = 290 K
0.36 × 0.20 × 0.16 mm

#### Data collection

Rigaku R-AXIS RAPID diffractometer
Absorption correction: multi-scan (*ABSCOR*; Higashi, 1995 ▶) *T*_min_ = 0.170, *T*_max_ = 0.309
12747 measured reflections
1550 independent reflections
1273 reflections with *I* > 2σ(*I*)
*R*_int_ = 0.090

#### Refinement

*R*[*F* ^2^ > 2σ(*F* ^2^)] = 0.042
*wR*(*F* ^2^) = 0.130
*S* = 1.14
1550 reflections
106 parameters
H-atom parameters constrained
Δρ_max_ = 0.84 e Å^−3^
Δρ_min_ = −0.78 e Å^−3^

### 

Data collection: *RAPID-AUTO* (Rigaku, 1998 ▶); cell refinement: *RAPID-AUTO*; data reduction: *CrystalStructure* (Rigaku, 1998 ▶); program(s) used to solve structure: *SHELXS97* (Sheldrick, 2008 ▶); program(s) used to refine structure: *SHELXL97* (Sheldrick, 2008 ▶); molecular graphics: *SHELXTL* (Sheldrick, 2008 ▶); software used to prepare material for publication: *SHELXL97*.

## Supplementary Material

Crystal structure: contains datablocks I, global. DOI: 10.1107/S1600536809045395/bh2255sup1.cif

Structure factors: contains datablocks I. DOI: 10.1107/S1600536809045395/bh2255Isup2.hkl

Additional supplementary materials:  crystallographic information; 3D view; checkCIF report

## Figures and Tables

**Table 1:** Hydrogen-bond geometry (Å, °)

*D*—H⋯*A*	*D*—H	H⋯*A*	*D*⋯*A*	*D*—H⋯*A*
O1—H1*A*⋯O2^i^	0.82	1.98	2.753 (5)	156
O1—H1*B*⋯Br1^ii^	0.82	2.81	3.603 (2)	164

Symmetry codes: (i) 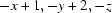; (ii) 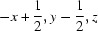.

**Fig. 1.**
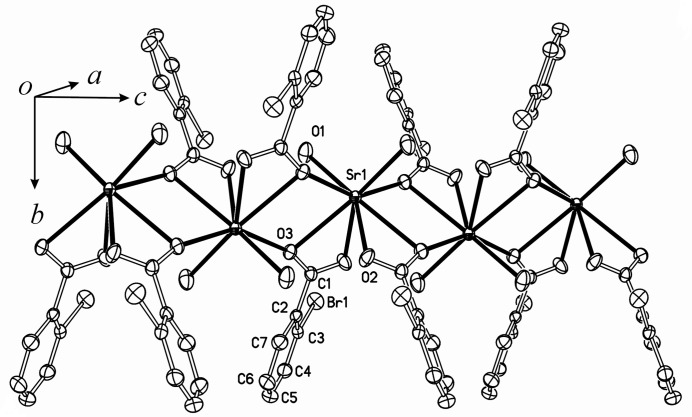
The one-dimensional chain structure of the title compound, showing the atom-labelling scheme. Displacement ellipsoids are drawn at the 50% probability level.

**Fig. 2.**
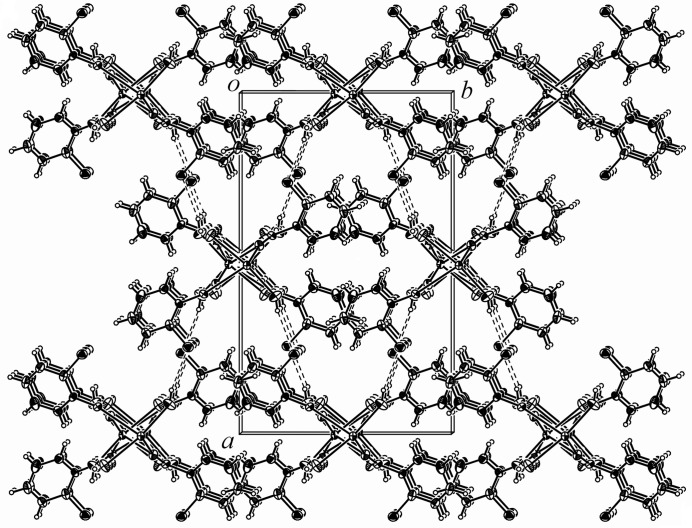
A packing diagram of the title complex, viewed along the c axis, The O—H···O and O—H···Br hydrogen bonds (dashed lines) in the title compound.

## supplementary crystallographic information

### Comment

Metal ions with 2-bromobenzoato ligands can form, among others, mononuclear, dinuclear complexes (Zhang *et al.*, 2005, 2008; Zhang, 2006; Wang *et al.*, 2003) but very few reports on one-dimensional chain structures complexes including 2-bromobenzoato ligands have been published.

In this paper, we would like to report the synthesis and crystal structure of a one-dimensional chain complex including 2-bromobenzoato and Strontium(II). The crystal structure of the title compound is similar to previously published structures (Zhang, 2008; Karipides *et al.*, 1988). Within the title compound, each Sr^II^ ion is located on a crystallographic two-fold axis and is coordinated by eight O atoms from two water molecules and four carboxyl groups of 2-bromobenzoic acid anions in an irregular coordination geometry. Two µ_3_-carboxyl groups of the 2-bromobenzoic anions bridge two symmetry related Strontium atoms, giving rise to a one-dimensional chain structure extending along the [001] direction, with Sr—O bond lengths in the range of 2.498 (3) to 2.753 (4) Å. Separation between Sr and Sr*^iv^* (symmetry code *iv*: -*x*+1, -*y*+2, -*z*+1) is 4.1703 (8) Å (Fig. 1). The polymeric chains are connected via O—H···O and O—H···Br hydrogen bonds interactions in a three-dimensional supramolecular structure (Fig. 2). The O1—H1A···O3 and O1—H1A···Br1 separations are 2.753 Å and 3.603 Å. The O—H···O and O1—H1A···Br1 bond angles are 156 ° and 164°, Table 2.

### Experimental

SrCl_2_.6H_2_O. (0.533 g, 2.00 mmol) was dissolved in the appropriate amount of water, and then 1M Na_2_CO_3_ solution was added. SrCO_3_ was obtained by filtration, which was then washed with distilled water (5 times). The freshly prepared SrCO_3_, 2-bromobenzoic acid (0.402 g, 2.00 mmol), CH_3_OH/H_2_O (*v*/*v* = 1:2, 15 ml) were mixed and stirred for 2.0 h. Subsequently, the resulting cream suspension was heated in a 23 ml Teflon-lined stainless steel autoclave at 433 K for 5800 minutes. After the autoclave was cooled to room temperature according to the procedure at 2600 minutes, the solid was filtered off. The resulting filtrate was allowed to stand at room temperature, and slow evaporation for 6 weeks afforded colorless block-shaped single crystals.

### Refinement

C-bound H atoms were placed in calculated positions, with C—H = 0.93Å and *U*_iso_(H) = 1.2*U*_eq_(C), and were refined using the riding- model approximation. The H atoms of the water molecule were located in a difference Fourier map and refined with an O—H distance restraint of 0.82 (1) Å and *U*_iso_(H) = 1.5*U*_eq_(O).

### Crystal data

**Table:**

[Sr(C_7_H_4_BrO_2_)_2_(H_2_O)_2_]	*F*(000) = 1008
*M**_r_* = 523.68	*D*_x_ = 1.975 Mg m^−^^3^
Orthorhombic, *P**b**c**n*	Mo *K*α radiation, λ = 0.71073 Å
Hall symbol: -P 2n 2ab	Cell parameters from 9800 reflections
*a* = 18.740 (4) Å	θ = 3.3–25.0°
*b* = 11.669 (2) Å	µ = 7.62 mm^−^^1^
*c* = 8.0529 (16) Å	*T* = 290 K
*V* = 1760.9 (6) Å^3^	Block, colorless
*Z* = 4	0.36 × 0.20 × 0.16 mm

### Data collection

**Table:**

Rigaku R-AXIS RAPID diffractometer	1550 independent reflections
Radiation source: fine-focus sealed tube	1273 reflections with *I* > 2σ(*I*)
graphite	*R*_int_ = 0.090
ω scans	θ_max_ = 25.0°, θ_min_ = 3.3°
Absorption correction: multi-scan (*ABSCOR*; Higashi, 1995)	*h* = −22→22
*T*_min_ = 0.170, *T*_max_ = 0.309	*k* = −13→13
12747 measured reflections	*l* = −9→8

### Refinement

**Table:**

Refinement on *F*^2^	Secondary atom site location: difference Fourier map
Least-squares matrix: full	Hydrogen site location: inferred from neighbouring sites
*R*[*F*^2^ > 2σ(*F*^2^)] = 0.042	H-atom parameters constrained
*wR*(*F*^2^) = 0.130	*w* = 1/[σ^2^(*F*_o_^2^) + (0.0652*P*)^2^ + 1.8313*P*] where *P* = (*F*_o_^2^ + 2*F*_c_^2^)/3
*S* = 1.14	(Δ/σ)_max_ < 0.001
1550 reflections	Δρ_max_ = 0.84 e Å^−^^3^
106 parameters	Δρ_min_ = −0.78 e Å^−^^3^
0 restraints	Extinction correction: *SHELXL97* (Sheldrick, 2008), Fc^*^=kFc[1+0.001xFc^2^λ^3^/sin(2θ)]^-1/4^
0 constraints	Extinction coefficient: 0.0016 (6)
Primary atom site location: structure-invariant direct methods	

### Fractional atomic coordinates and isotropic or equivalent isotropic displacement parameters (Å^2^)

**Table:**

	*x*	*y*	*z*	*U*_iso_*/*U*_eq_	
Sr1	0.5000	0.95347 (6)	0.2500	0.0294 (3)	
Br1	0.26073 (4)	1.23028 (6)	0.28032 (9)	0.0528 (3)	
O1	0.4082 (2)	0.8205 (4)	0.1116 (5)	0.0577 (12)	
H1A	0.4050	0.8178	0.0101	0.087*	
H1B	0.3663	0.8112	0.1371	0.087*	
O2	0.5875 (3)	1.1245 (4)	0.2207 (4)	0.0523 (13)	
O3	0.4433 (2)	1.1017 (3)	0.0190 (4)	0.0395 (10)	
C1	0.4152 (3)	1.1591 (4)	0.1317 (6)	0.0335 (12)	
C2	0.3870 (3)	1.2758 (4)	0.0915 (6)	0.0318 (12)	
C3	0.3238 (3)	1.3201 (4)	0.1490 (6)	0.0404 (14)	
C4	0.3005 (4)	1.4307 (5)	0.1105 (8)	0.0475 (16)	
H4	0.2569	1.4575	0.1495	0.057*	
C5	0.3434 (5)	1.4993 (5)	0.0138 (8)	0.0549 (19)	
H5A	0.3293	1.5739	−0.0105	0.066*	
C6	0.4068 (5)	1.4582 (5)	−0.0469 (8)	0.060 (2)	
H6	0.4352	1.5050	−0.1129	0.072*	
C7	0.4288 (3)	1.3487 (5)	−0.0113 (7)	0.0452 (15)	
H7	0.4716	1.3218	−0.0549	0.054*	

### Atomic displacement parameters (Å^2^)

**Table:**

	*U*^11^	*U*^22^	*U*^33^	*U*^12^	*U*^13^	*U*^23^
Sr1	0.0387 (5)	0.0285 (4)	0.0210 (4)	0.000	0.0023 (3)	0.000
Br1	0.0460 (5)	0.0583 (5)	0.0542 (5)	0.0021 (3)	0.0062 (3)	0.0062 (3)
O1	0.058 (3)	0.077 (3)	0.038 (2)	−0.029 (2)	−0.002 (2)	0.003 (2)
O2	0.076 (4)	0.053 (3)	0.028 (2)	−0.026 (2)	0.007 (2)	−0.0068 (18)
O3	0.052 (3)	0.041 (2)	0.0251 (19)	0.0051 (18)	0.0075 (17)	−0.0051 (16)
C1	0.034 (3)	0.039 (3)	0.028 (3)	0.007 (2)	0.000 (2)	−0.001 (2)
C2	0.037 (3)	0.031 (3)	0.027 (3)	0.008 (2)	−0.003 (2)	−0.006 (2)
C3	0.060 (4)	0.035 (3)	0.026 (3)	0.008 (3)	−0.010 (3)	0.000 (2)
C4	0.053 (4)	0.043 (3)	0.047 (4)	0.011 (3)	−0.009 (3)	−0.006 (3)
C5	0.080 (6)	0.034 (3)	0.052 (4)	0.010 (3)	−0.011 (4)	0.001 (3)
C6	0.087 (6)	0.046 (4)	0.047 (4)	−0.014 (4)	−0.006 (4)	0.012 (3)
C7	0.047 (4)	0.044 (3)	0.045 (3)	−0.005 (3)	0.004 (3)	0.000 (3)

### Geometric parameters (Å, °)

**Table:**

Sr1—O3^i^	2.498 (3)	O3—C1	1.244 (6)
Sr1—O3^ii^	2.498 (3)	O3—Sr1^i^	2.498 (3)
Sr1—O1	2.570 (4)	C1—O2^iii^	1.257 (6)
Sr1—O1^iii^	2.570 (4)	C1—C2	1.496 (7)
Sr1—O2	2.594 (4)	C2—C3	1.373 (8)
Sr1—O2^iii^	2.594 (4)	C2—C7	1.422 (8)
Sr1—O3^iii^	2.753 (4)	C3—C4	1.397 (8)
Sr1—O3	2.753 (4)	C4—C5	1.376 (10)
Sr1—C1^iii^	3.031 (5)	C4—H4	0.9300
Sr1—C1	3.031 (5)	C5—C6	1.371 (11)
Br1—C3	1.901 (6)	C5—H5A	0.9300
O1—H1A	0.8200	C6—C7	1.373 (9)
O1—H1B	0.8200	C6—H6	0.9300
O2—C1^iii^	1.257 (6)	C7—H7	0.9300
			
O3^i^—Sr1—O3^ii^	150.14 (16)	O2—Sr1—Sr1^iv^	83.55 (8)
O3^i^—Sr1—O1	75.71 (13)	O2^iii^—Sr1—Sr1^iv^	73.25 (8)
O3^ii^—Sr1—O1	86.32 (12)	O3^iii^—Sr1—Sr1^iv^	35.34 (7)
O3^i^—Sr1—O1^iii^	86.32 (12)	O3—Sr1—Sr1^iv^	119.25 (7)
O3^ii^—Sr1—O1^iii^	75.71 (13)	C1^iii^—Sr1—Sr1^iv^	59.36 (10)
O1—Sr1—O1^iii^	105.7 (2)	C1—Sr1—Sr1^iv^	95.59 (10)
O3^i^—Sr1—O2	81.37 (12)	O3^i^—Sr1—Sr1^i^	39.61 (8)
O3^ii^—Sr1—O2	123.12 (11)	O3^ii^—Sr1—Sr1^i^	154.76 (9)
O1—Sr1—O2	147.99 (12)	O1—Sr1—Sr1^i^	74.84 (9)
O1^iii^—Sr1—O2	94.65 (16)	O1^iii^—Sr1—Sr1^i^	125.16 (9)
O3^i^—Sr1—O2^iii^	123.12 (11)	O2—Sr1—Sr1^i^	73.25 (8)
O3^ii^—Sr1—O2^iii^	81.37 (12)	O2^iii^—Sr1—Sr1^i^	83.55 (8)
O1—Sr1—O2^iii^	94.65 (16)	O3^iii^—Sr1—Sr1^i^	119.25 (7)
O1^iii^—Sr1—O2^iii^	147.99 (12)	O3—Sr1—Sr1^i^	35.34 (7)
O2—Sr1—O2^iii^	79.4 (2)	C1^iii^—Sr1—Sr1^i^	95.59 (10)
O3^i^—Sr1—O3^iii^	125.68 (15)	C1—Sr1—Sr1^i^	59.36 (9)
O3^ii^—Sr1—O3^iii^	74.95 (13)	Sr1^iv^—Sr1—Sr1^i^	149.82 (4)
O1—Sr1—O3^iii^	158.51 (13)	Sr1—O1—H1A	120.4
O1^iii^—Sr1—O3^iii^	80.09 (12)	Sr1—O1—H1B	127.8
O2—Sr1—O3^iii^	48.25 (10)	H1A—O1—H1B	99.9
O2^iii^—Sr1—O3^iii^	72.51 (13)	C1^iii^—O2—Sr1	97.8 (3)
O3^i^—Sr1—O3	74.95 (13)	C1—O3—Sr1^i^	162.0 (3)
O3^ii^—Sr1—O3	125.68 (15)	C1—O3—Sr1	90.5 (3)
O1—Sr1—O3	80.09 (12)	Sr1^i^—O3—Sr1	105.05 (13)
O1^iii^—Sr1—O3	158.51 (13)	O3—C1—O2^iii^	122.3 (5)
O2—Sr1—O3	72.51 (13)	O3—C1—C2	118.8 (4)
O2^iii^—Sr1—O3	48.25 (10)	O2^iii^—C1—C2	118.9 (4)
O3^iii^—Sr1—O3	102.18 (15)	O3—C1—Sr1	65.3 (3)
O3^i^—Sr1—C1^iii^	104.69 (13)	O2^iii^—C1—Sr1	58.0 (3)
O3^ii^—Sr1—C1^iii^	98.88 (13)	C2—C1—Sr1	166.8 (4)
O1—Sr1—C1^iii^	164.74 (14)	C3—C2—C7	116.5 (5)
O1^iii^—Sr1—C1^iii^	89.49 (15)	C3—C2—C1	125.1 (5)
O2—Sr1—C1^iii^	24.26 (12)	C7—C2—C1	118.4 (5)
O2^iii^—Sr1—C1^iii^	72.16 (16)	C2—C3—C4	122.8 (6)
O3^iii^—Sr1—C1^iii^	24.23 (11)	C2—C3—Br1	121.1 (4)
O3—Sr1—C1^iii^	85.27 (13)	C4—C3—Br1	116.0 (5)
O3^i^—Sr1—C1	98.88 (13)	C5—C4—C3	118.7 (6)
O3^ii^—Sr1—C1	104.69 (13)	C5—C4—H4	120.6
O1—Sr1—C1	89.49 (15)	C3—C4—H4	120.6
O1^iii^—Sr1—C1	164.74 (14)	C6—C5—C4	120.2 (6)
O2—Sr1—C1	72.16 (16)	C6—C5—H5A	119.9
O2^iii^—Sr1—C1	24.26 (12)	C4—C5—H5A	119.9
O3^iii^—Sr1—C1	85.27 (13)	C5—C6—C7	120.8 (7)
O3—Sr1—C1	24.23 (11)	C5—C6—H6	119.6
C1^iii^—Sr1—C1	75.3 (2)	C7—C6—H6	119.6
O3^i^—Sr1—Sr1^iv^	154.76 (9)	C6—C7—C2	120.8 (6)
O3^ii^—Sr1—Sr1^iv^	39.61 (8)	C6—C7—H7	119.6
O1—Sr1—Sr1^iv^	125.16 (9)	C2—C7—H7	119.6
O1^iii^—Sr1—Sr1^iv^	74.84 (9)		

Symmetry codes: (i) −*x*+1, −*y*+2, −*z*; (ii) *x*, −*y*+2, *z*+1/2; (iii) −*x*+1, *y*, −*z*+1/2; (iv) −*x*+1, −*y*+2, −*z*+1.

### Hydrogen-bond geometry (Å, °)

**Table:**

*D*—H···*A*	*D*—H	H···*A*	*D*···*A*	*D*—H···*A*
O1—H1A···O2^i^	0.82	1.98	2.753 (5)	156
O1—H1B···Br1^v^	0.82	2.81	3.603 (2)	164

Symmetry codes: (i) −*x*+1, −*y*+2, −*z*; (v) −*x*+1/2, *y*−1/2, *z*.
